# Fatal Retention of Urine

**Published:** 1831-04-01

**Authors:** 


FATAL RETENTION OF URINE.

M. CAMBOURNAC relates the following case in a late Number of the Journal Hebdomadaire.

*Case.* F. Perrot, aged 28 years, of robust constitution, lived an irregular life, and was often subject to retentions of urine. In December 1829, the weather being very severe, he was affected with hæmaturia and retention, and our author could not introduce the catheter, in consequence of a stricture about the middle of the urethra. A conical bougie was made to bear on the obstruction, and leeches were applied to the perineum, in addition to semicupia and diluent drinks. In three days the urine flowed by the natural channel, and the patient prematurely resumed his avocations. In June of 1830, M. C. was again summoned, in consequence of complete retention. By the same means, the urine was again made to flow. On the 15th of August, this man acted as one of the National Guards, and was much exposed to rain—after which he supped at a restaurateur's, but did not commit any excess. The same evening he was seized with dysuria, and violent pain in the region of the bladder. He could not pass a drop of water. Leeches were applied, and various means were used; but with little success. On the 18th the constitutional symptoms assumed an alarming character, and the brain was evidently suffering. The abdomen was distended, and the hypogastrium tense and hard. Puncture of the bladder above the pubes was now performed, *and a little to the left of the* median line. No water flowed on withdrawing the stillette. He died the next day.

*Dissection.* The peritoneum was much injected; but there was very little fluid effused into the cavity. The fundus of the bladder projected about an inch above the *right* brim of the pubis. The urethra presented an impenetrable stricture. The bladder was very little distended, and the trocar had only grazed the fundus, without penetrating into its cavity. Each ureter was the size of the intestine of a fowl, distended by urine, and the coats almost transparent. The left kidney was much enlarged, and its pelvis distended by urine. The pelvis of the right kidney was also enormously distended with urine. No obstruction appears to have been found to the course of the urine from the kidneys to the bladder—and the trifling distention of the bladder itself is, to us, an inexplicable phenomenon. We cannot conceive the reason why the author deviated from the middle space in making his puncture above the pubes; and altogether we look upon the operation as a very left-handed kind of business, and the dissection as any thing but satisfactory.

